# Dengue Virus Co-opts UBR4 to Degrade STAT2 and Antagonize Type I Interferon Signaling

**DOI:** 10.1371/journal.ppat.1003265

**Published:** 2013-03-28

**Authors:** Juliet Morrison, Maudry Laurent-Rolle, Ana M. Maestre, Ricardo Rajsbaum, Giuseppe Pisanelli, Viviana Simon, Lubbertus C. F. Mulder, Ana Fernandez-Sesma, Adolfo García-Sastre

**Affiliations:** 1 Department of Microbiology, Icahn School of Medicine at Mount Sinai, New York, New York, United States of America; 2 Global Health and Emerging Pathogens Institute, Icahn School of Medicine at Mount Sinai, New York, New York, United States of America; 3 Department of Medicine, Icahn School of Medicine at Mount Sinai, New York, New York, United States of America; 4 Department of Veterinary Medicine and Animal Production, University of Naples Federico II, Naples, Italy; Washington University School of Medicine, United States of America

## Abstract

An estimated 50 million dengue virus (DENV) infections occur annually and more than forty percent of the human population is currently at risk of developing dengue fever (DF) or dengue hemorrhagic fever (DHF). Despite the prevalence and potential severity of DF and DHF, there are no approved vaccines or antiviral therapeutics available. An improved understanding of DENV immune evasion is pivotal for the rational development of anti-DENV therapeutics. Antagonism of type I interferon (IFN-I) signaling is a crucial mechanism of DENV immune evasion. DENV NS5 protein inhibits IFN-I signaling by mediating proteasome-dependent STAT2 degradation. Only proteolytically-processed NS5 can efficiently mediate STAT2 degradation, though both unprocessed and processed NS5 bind STAT2. Here we identify UBR4, a 600-kDa member of the N-recognin family, as an interacting partner of DENV NS5 that preferentially binds to processed NS5. Our results also demonstrate that DENV NS5 bridges STAT2 and UBR4. Furthermore, we show that UBR4 promotes DENV-mediated STAT2 degradation, and most importantly, that UBR4 is necessary for efficient viral replication in IFN-I competent cells. Our data underscore the importance of NS5-mediated STAT2 degradation in DENV replication and identify UBR4 as a host protein that is specifically exploited by DENV to inhibit IFN-I signaling via STAT2 degradation.

## Author Summary

Dengue virus (DENV) is the leading cause of mosquito-borne viral illness and death in humans. At present, there are no vaccines and no specific antiviral therapeutics to prevent or treat DENV infections. We previously described that the NS5 protein of DENV inhibits type I interferon signaling in virus-infected cells by mediating STAT2 degradation. This property allows DENV to overcome the antiviral effects of type I interferon, contributing to viral replication in the host. We have now obtained new insight into the mechanism by which DENV NS5 induces STAT2 degradation. NS5 bridges STAT2 with the cellular protein UBR4, a member of a family of predicted E3 ligases, resulting in UBR4-mediated STAT2 degradation. Elimination of UBR4 or mutations in NS5 that prevent its binding to UBR4 prevent NS5 from inducing STAT2 degradation. Importantly, UBR4 is required for optimal DENV replication in the presence of a competent type I interferon system. Our data demonstrate the requirement of a host factor, UBR4, for DENV to overcome the antiviral interferon response. This information might be important for the design of specific DENV inhibitors that prevent dengue virus from evading innate immunity.

## Introduction

Approximately fifty million dengue virus (DENV) infections occur annually with more than forty percent of the human population at risk of developing dengue fever (DF) or dengue hemorrhagic fever (DHF). DF and its more severe form, DHF, are potentially fatal diseases caused by the four serotypes (1, 2, 3 and 4) of DENV [1]. As no approved vaccines or antiviral therapeutics are available for the prevention or treatment of DENV infections, it is imperative that the biology and immunology of DENV infections are better understood. An in depth comprehension of DENV-host interactions will accelerate our progress in developing DENV therapeutics.

DENV, along with other clinically relevant arboviruses such as West Nile virus (WNV), Japanese encephalitis virus (JEV) and yellow fever virus (YFV), belongs to the flavivirus genus of the *Flaviviridae* family. The flavivirus genome is a capped 11 kb genome that is translated into a single polyprotein, which is cleaved both by the viral protease (NS2B/3) and host proteases to yield three structural proteins (capsid [C], membrane [prM/M] and envelop [E]) and seven non-structural proteins (NS1, NS2A, NS2B, NS3, NS4A, NS4B and NS5) [2], [3]. The flavivirus structural proteins incorporate the viral genome into newly generated virions while the non-structural proteins replicate the viral genome and exploit the cellular machinery to subvert host immune responses. The approximately 900-amino-acid NS5 protein is the largest and most conserved flavivirus protein [4]. This multifunctional protein has RNA-dependent RNA polymerase (RdRp) activity as well as methyltransferase activity [5], [6], [7], [8], [9]. In addition, more recent studies have shown that NS5 is a potent interferon-signaling antagonist [10], [11], [12], [13], [14], [15], [16].

The significance of the interferon (IFN) response as an important component of host immunity is underscored by numerous examples of viruses that antagonize it [17], [18], [19], [20], [21], [22], [23], [24], [25]. Viruses express pathogen-associated molecular patterns (PAMPs) that trigger the production of type I IFN (IFNα/β or IFN-I) [26]. Binding of IFN-I to the cell-surface IFN-I receptor (IFNAR) initiates a signaling cascade that results in the activation and phosphorylation of the Janus kinases, Jak1 and Tyk2, and the transcription factors, STAT1 and STAT2. Phosphorylated STAT1 and STAT2 along with IRF9 form the heterotrimeric transcriptional complex, ISGF3 [27], [28], and induce the expression of antiviral IFN-stimulated genes (ISGs) [29], [30], [31], [32].

DENV encodes several antagonists of both IFN-I production and IFN-I signaling [13], [14], [33], [34], [35], [36], [37], [38]. The NS5 proteins of DENV and other flaviviruses have been shown to be potent inhibitors of IFN signaling. NS5 proteins of different flaviviruses may target different steps of the IFN signaling pathway. For example, WNV NS5 prevents the phosphorylation of the STAT proteins, while DENV NS5 binds human STAT2 and promotes its proteasomal degradation [13], [15].

Although STAT degradation is a common mechanism of viral IFN antagonism [19], [20], [39], [40], the requirements for DENV NS5-mediated STAT2 degradation are unique. DENV NS5-mediated STAT2 degradation requires NS5 to be proteolytically cleaved at its N terminus from a larger precursor protein [13]. N-terminal cleavage of NS5 normally occurs during DENV infection because the NS2B/3 protease cleaves at the junction located between NS4B and NS5 thereby releasing NS5 from the viral polyprotein [41]. Though both unprocessed and proteolytically-processed NS5 can bind STAT2, only processed NS5 can efficiently mediate STAT2 degradation [13]. Furthermore, the first ten amino acids of NS5 are dispensable for STAT2 binding but are indispensable for STAT2 degradation [13]. While the viral requirements for DENV-mediated STAT2 degradation are known, the cellular components were unspecified until now.

This study identifies the 600-kDa protein, UBR4, as a binding partner of DENV NS5. UBR4 is a member of the N-recognin family, which contains proven and predicted E3 ligases that recognize and degrade proteins containing destabilizing N termini [42]. UBR4 interacts preferentially with proteolytically-processed DENV NS5 but not with YFV NS5 or WNV NS5, highlighting the specificity of the DENV NS5/UBR4 interaction. Furthermore, we have identified two residues within the first 10 N-terminal amino acids of NS5, threonine 2 and glycine 3, that are required for NS5 binding to UBR4. These two residues are conserved across the four DENV serotypes but are not found in other flaviviruses. Finally, we show that UBR4 is required for DENV-mediated STAT2 degradation, and for efficient DENV replication in IFN-I competent cells. Our data confirm the importance of NS5-mediated STAT2 degradation for DENV replication, and identify UBR4 as a host protein that is specifically co-opted by DENV to inhibit IFN-I signaling via STAT2 degradation.

## Results

### DENV NS5 binds UBR4

**Figure 1:**
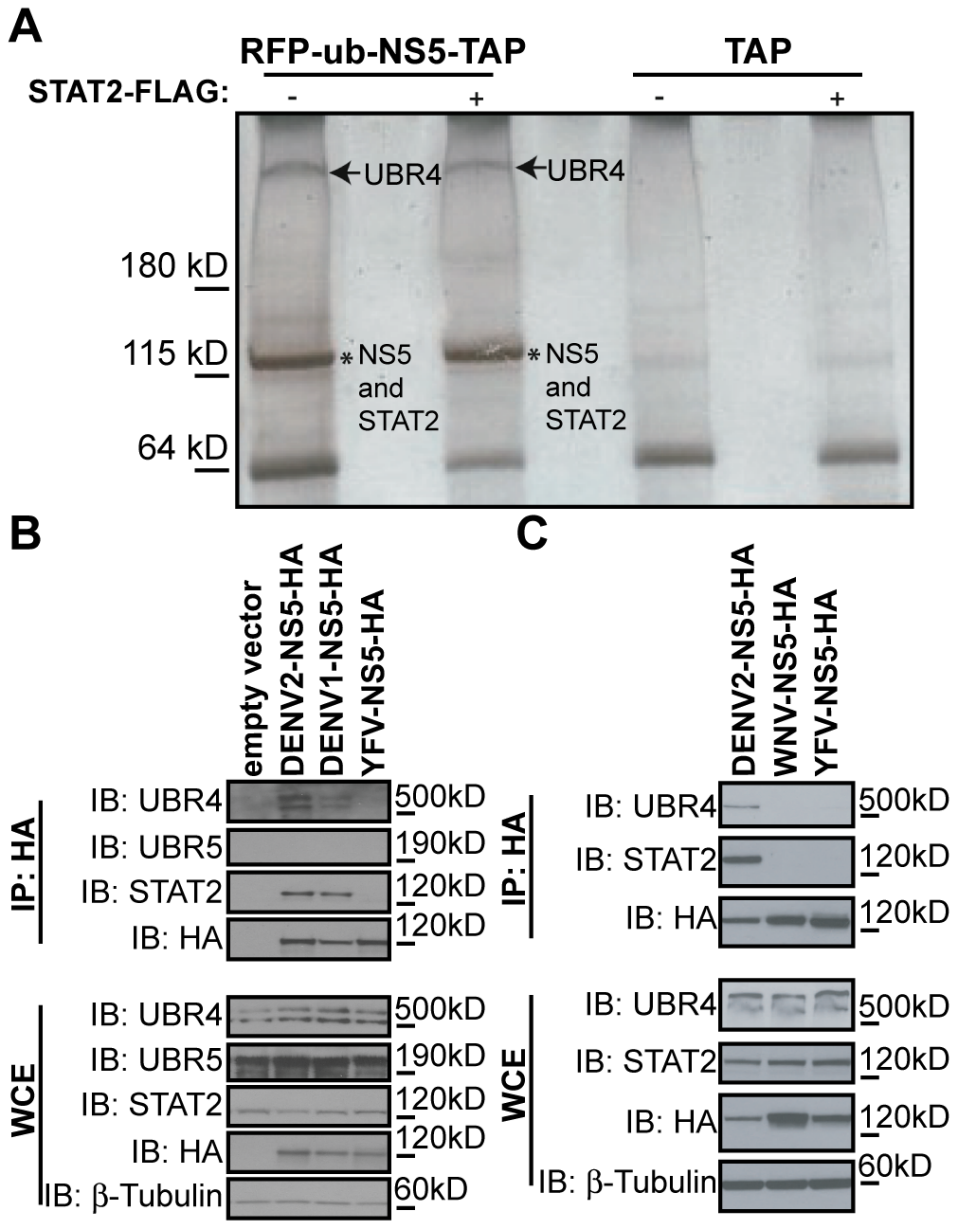
DENV NS5 binds UBR4. (A) RFP-ubiquitin-NS5-TAP was expressed in 293T cells, in the presence or absence of human STAT2-FLAG, and then purified using the tandem affinity purification (TAP) method. The purified proteins were resolved on an SDS 4–15% gel that was then stained with silver. *NS5 and STAT2 run in similar positions in the gel. The bands highlighted by the arrows were analyzed by mass spectrometry at Taplin Biological Mass Spectrometry Facility, Harvard Medical School. (B and C) HA-tagged DENV2, DENV1, WNV and/or YFV NS5 proteins were immunoprecipitated from 293T cells using anti-HA beads. Immunoblot (IB) analysis of the immunoprecipitate (IP) and whole cell extract (WCE) was performed against UBR4, UBR5, STAT2, HA and/or β-tubulin.

DENV NS5 binds human and non-human primate STAT2 but cannot efficiently mediate STAT2 degradation unless it is expressed in the context of a precursor protein from which it is N-terminally cleaved [13]. When DENV NS5 is engineered to be expressed downstream of ubiquitin, cellular hydrolases cleave ubiquitin in a manner that mimics the cleavage of NS4B away from NS5 by the NS2B/3 protease during DENV infection [13]. To identify host proteins that are required for NS5-mediated degradation of STAT2, we generated a DENV2 NS5 construct consisting of RFP-ubiquitin fused to the NS5 N-terminus and a TAP (tandem-affinity purification) tag fused to the NS5 C-terminus. This NS5 construct was expressed in 293T cells, in the presence or absence of human STAT2-FLAG, and then purified using the TAP method. A high molecular weight protein band was consistently and specifically co-precipitated with NS5 both in the presence and absence of overexpressed STAT2 (Figure 1A). Trypsin digestion of this band yielded five peptides that were identified by mass spectrometry as sequences of the N-recognin, UBR4 (Table 1) [42]. To confirm the binding and specificity of the interaction between DENV2 NS5 and UBR4, HA-tagged DENV2 NS5, DENV1 NS5, and YFV NS5 were expressed in 293T cells and purified by immunoprecipitation with antibodies raised against the HA epitope. The NS5 protein of both DENV1 and DENV2 precipitated UBR4 from 293T cells but YFV NS5 was unable to precipitate UBR4 from these cells (Figure 1B). WNV NS5 was also unable to bind UBR4 (Figure 1C). In order to assess the specificity of DENV NS5 for UBR4, we also examined the ability of NS5 to bind another member of the N-recognin family, UBR5. DENV1 and DENV2 NS5 bound UBR4 but not UBR5, further highlighting the unique interaction between UBR4 and DENV NS5 (Figure 1B).

**Table 1:** UBR4 peptides identified by mass spectrometry.

Peptide sequence	Position in UBR4 protein (NCBI Accession # Q5T4S7)
RISESLVRH	1750–1758
KSFAATISRT	2130–2139
RIQIGTQAIERA	2353–2364
RALATNPALRH	3888–3898
RQVLFTPATQAARQ	4130–4143

### The UBR4-NS5 interaction correlates with NS5-mediated STAT2 degradation

**Figure 2:**
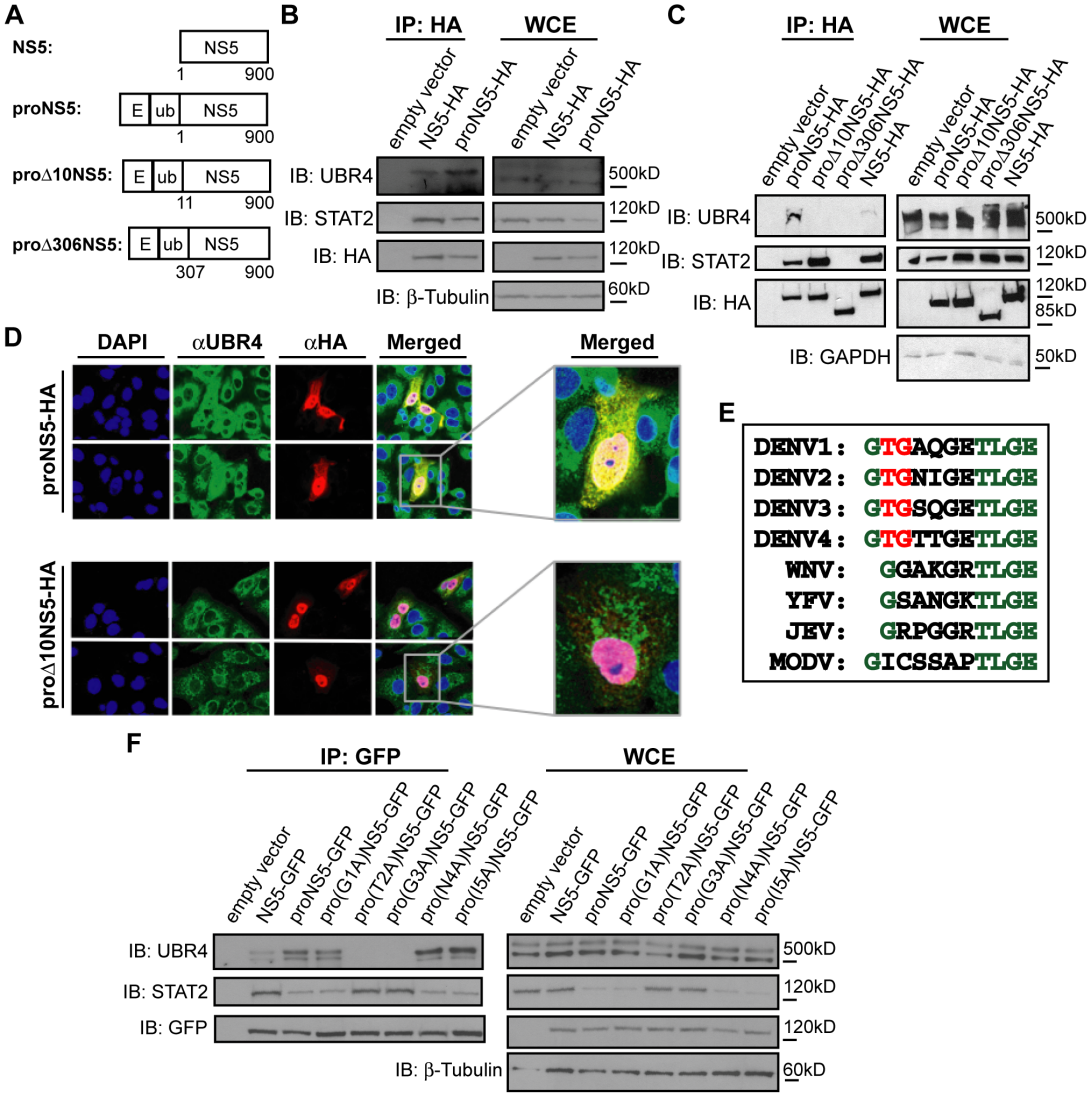
The UBR4-NS5 interaction correlates with NS5-mediated STAT2 degradation. (A) A representation of the DENV2 NS5 constructs used in this study. The E domain upstream of ubiquitin refers to the E protein of DENV2. (B and C) HA-tagged DENV NS5 constructs were immunoprecipitated from 293T cells using anti-HA beads. Immunoblot (IB) analysis of the immunoprecipitate (IP) and whole cell extract (WCE) was performed against UBR4, STAT2, HA and β-tubulin or GAPDH. (D) Vero cells expressing HA-tagged DENV NS5 constructs were fixed for immunofluorescence (IF) analysis of UBR4 (green) and NS5 (red). The nuclei were also stained with DAPI (blue). (E) An alignment of the first 10 or 11 amino acids of NS5 from several flaviviruses. Residues that are conserved in all examined flaviviruses are highlighted in green, while those residues that are only conserved amongst the four DENV serotypes are highlighted in red. (F) GFP-tagged DENV2 NS5 constructs and DENV2 NS5 point mutants were immunoprecipitated from 293T cells using anti-GFP antibody and protein G beads. Immunoblot (IB) analysis of the immunoprecipitate (IP) and whole cell extract (WCE) was performed against UBR4, STAT2, GFP and β-tubulin.

UBR4 was found to bind both processed (RFP-ubiquitin-NS5-TAP) (Figure 1A), as well as unprocessed NS5 (NS5-HA) (Figure 1B). Since cleavage of NS5 promotes STAT2 degradation, we tested whether proteolytic processing would also affect the binding efficiency of NS5 for UBR4. The E domain in Figure 2A refers to the E protein of DENV2. Inclusion of a protein (such as E or RFP) upstream of ubiquitin allows one to differentiate between cleaved and uncleaved NS5 [13]. HA-tagged unprocessed NS5 (NS5-HA) and processed NS5 (proNS5-HA) (Figure 2A) were expressed in 293T cells and the NS5 proteins were immunoprecipitated and tested for UBR4 binding. Although both constructs precipitated UBR4, proNS5-HA precipitated two-fold higher amounts of UBR4 than NS5-HA did (as quantified by densitometry) (Figure 2B).

Given that the first 10 amino acids of NS5 are dispensable for STAT2 binding but indispensable for STAT2 degradation [13], we asked whether this N terminal region of NS5 was also important for UBR4 binding. To test this, we expressed and immunoprecipitated processed HA-tagged DENV NS5 proteins containing a deletion of 10 or 306 residues at their N-termini, and assessed their ability to bind UBR4. Full length HA-tagged NS5 (NS5-HA) was able to precipitate UBR4 and STAT2, and its ability to precipitate UBR4, but not STAT2, was increased seven-fold (as quantified by densitometry) when DENV NS5 was proteolytically-processed (proNS5-HA) (Figure 2C). Proteolytically-processed NS5 lacking the first 10 amino acids (proΔ10NS5-HA) precipitated STAT2 but not UBR4, and proteolytically-processed NS5 lacking the first 306 amino acids (proΔ306NS5-HA) precipitated neither protein (Figure 2C). When protein levels were examined in the whole cell extracts (WCE), STAT2 was reduced in proNS5-HA-expressing cells and slightly reduced in NS5-HA-expressing cells compared with proΔ10NS5-HA- or proΔ306NS5-HA-expressing cells (Figure 2C), which is consistent with published reports [13]. Thus, only the NS5 proteins that bound UBR4 could mediate STAT2 degradation, and increased UBR4 binding by NS5 correlated with increased NS5-mediated STAT2 degradation. The interaction of NS5 with UBR4 and the requirement for the first 10 amino acids of NS5 in mediating this DENV NS5-UBR4 interaction was also observed by NS5-UBR4 colocalization using immunofluorescence analysis in Vero cells (Figure 2D). To further define which of the N-terminal residues of DENV NS5 are required for its interaction with UBR4, alanine scanning of the first 5 amino acids of DENV NS5 was conducted (Figure 2E). Immunoprecipitation experiments with these mutant proteins revealed that the threonine (T) at position 2 and the glycine (G) at position 3, which are conserved among the DENV serotypes but absent in other flaviviruses, were required for NS5/UBR4 interaction and NS5-mediated STAT2 degradation (Figure 2F).

### NS5 interaction with UBR4 is independent of STAT2

**Figure 3:**
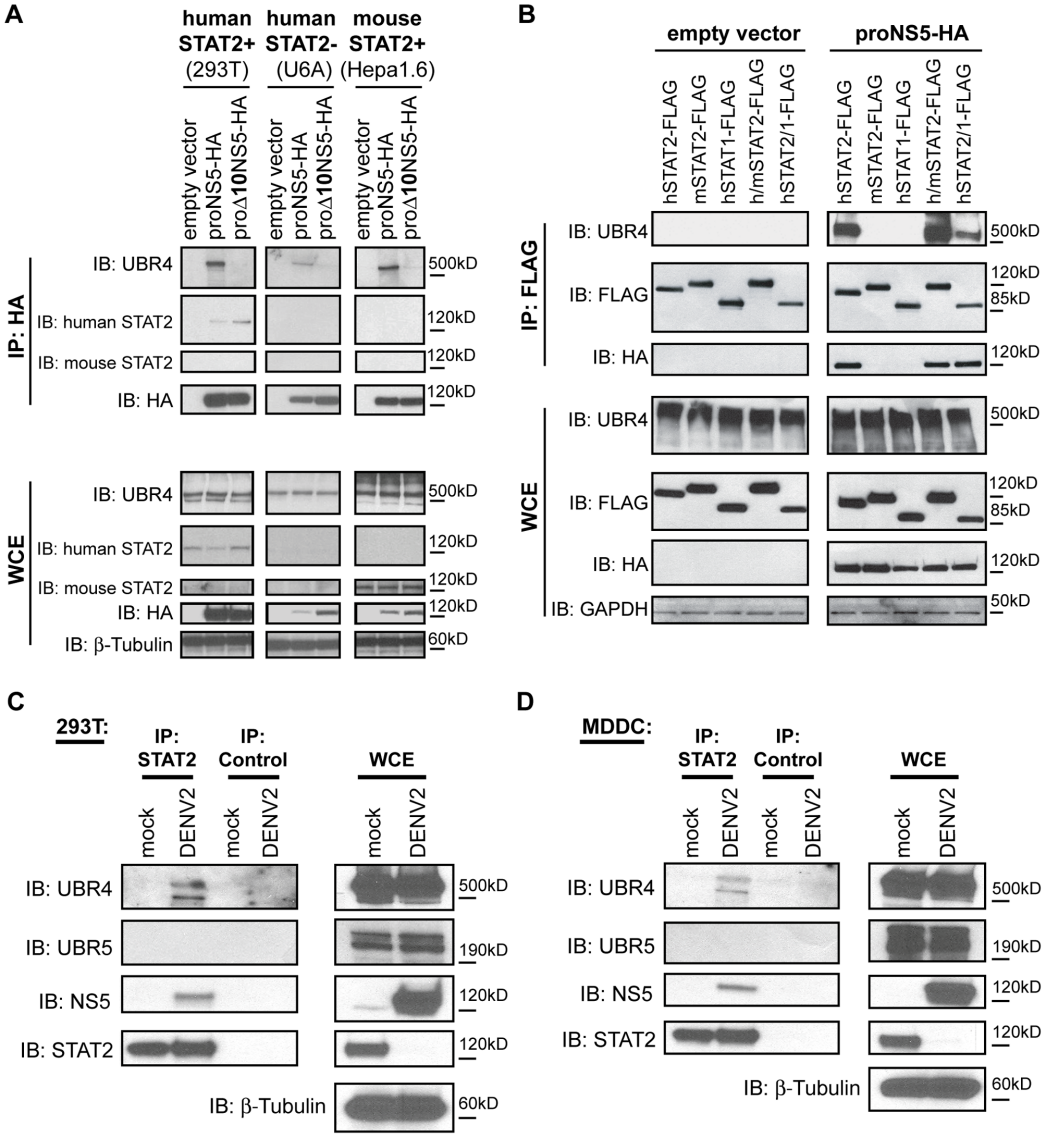
NS5 promotes STAT2 association with UBR4. (A) Proteolytically-processed HA-tagged DENV2 NS5 constructs or an empty vector were expressed in human cells that naturally express human STAT2 (293T cells), human cells that do not express STAT2 (U6A cells), or murine cells that naturally express mouse STAT2 (Hepa1.6), and the lysates of these cells were subjected to anti-HA immunoprecipitation. Immunoblot (IB) analysis of the immunoprecipitate (IP) and whole cell extract (WCE) was performed against UBR4, human STAT2, mouse STAT2, HA and β-tubulin. (B) FLAG-tagged STAT constructs were overexpressed in 293T cells with or without proteolytically-processed HA-tagged DENV2 NS5. Immunoprecipitation was performed against the FLAG epitope, then IB analysis of the IP and WCE was performed against UBR4, FLAG, HA and GAPDH. hSTAT2 = human STAT2; mSTAT2 = mouse STAT2; hSTAT1 = human STAT1; h/mSTAT2 = a chimeric protein with the first 301 amino acids of mouse STAT2 replaced by the corresponding human STAT2 sequence; hSTAT2/1 = a chimeric protein with the first 316 of human STAT1 amino acids replaced by the corresponding human STAT2 sequence. (C) 293T cells and (D) monocyte-derived dendritic cells (MDDC) were mock infected or infected with DENV2 (Thailand/16681 strain) at an MOI of 3 for 24 hours then lysed for IP with anti-STAT2 antibody or control IgG. IB analysis of the IP and WCE was performed against UBR4, UBR5, STAT2, DENV NS5 and β-tubulin.

The fact that NS5 mutants lacking residues T2 or G3 bound STAT2 but not UBR4 (Figure 2F) suggested that the interaction between NS5 and UBR4 was independent of STAT2. To confirm this result, the STAT2-deficient U6A cell line [43] was transfected with proNS5-HA or proΔ10NS5-HA. ProNS5-HA, but not proΔ10NS5-HA, precipitated UBR4 from U6A cells (Figure 3A). Unlike with human STAT2 (hSTAT2), NS5 does not bind and subsequently degrade mouse STAT2 (mSTAT2) [44]. The STAT2 proteins of mouse and human are divergent and share only 70% identity but the UBR4 proteins of mouse and human are 97% identical. When proNS5-HA was expressed in mouse cells (Hepa1.6), mouse UBR4 bound proNS5-HA but not proΔ10NS5-HA confirming that an interaction between NS5 and STAT2 is not required for NS5 to interact with UBR4 (Figure 3A). These data suggest that NS5 requires binding to both UBR4 and STAT2 to mediate STAT2 degradation.

### Human STAT2 forms a complex with UBR4 in the presence of DENV NS5

NS5 binds the coiled-coil region located within the first half of hSTAT2 [44]. Though mSTAT2 and human STAT1 (hSTAT1) cannot bind NS5, chimeric proteins that replace the first 301 amino acids of mSTAT2 (h/mSTAT2) or the first 316 amino acids of hSTAT1 (hSTAT2/1) with those of hSTAT2 can bind NS5 [44]. We expressed and immunoprecipitated FLAG-tagged STAT proteins and STAT chimeric proteins in the presence or absence of proNS5-HA. When hSTAT2 was overexpressed, STAT2 degradation was not observed because STAT2 degradation was likely masked by the large amount of overexpressed STAT2 present (Figure 3B). However, we observed that while hSTAT1 and mSTAT2 did not bind UBR4, h/mSTAT2, hSTAT2/1 and hSTAT2 all bound UBR4 in the presence of proNS5-HA (Figure 3B). Since only those STAT molecules that could bind NS5 could also bind UBR4, and NS5 binds UBR4 in the absence of STAT2, we conclude that NS5 serves as a bridge molecule between STAT2 and UBR4.

These results were confirmed in the context of DENV infection of a transformed human cell line (293T) and primary untransformed human cells (monocyte-derived dendritic cells or MDDCs) (Figure 3C and 3D). Cells were infected with DENV2 at an MOI of 3 for 24 hours, and lysed for immunoprecipitation with STAT2 antibodies or control IgG. Although the majority of STAT2 was degraded during DENV infection, the remaining STAT2 co-immunoprecipitated UBR4 from DENV-infected cells but not from mock-infected cells (Figure 3C and 3D), which is consistent with NS5 binding to and bringing together STAT2 and UBR4 during DENV infection.

### UBR4 promotes DENV-mediated STAT2 degradation

**Figure 4:**
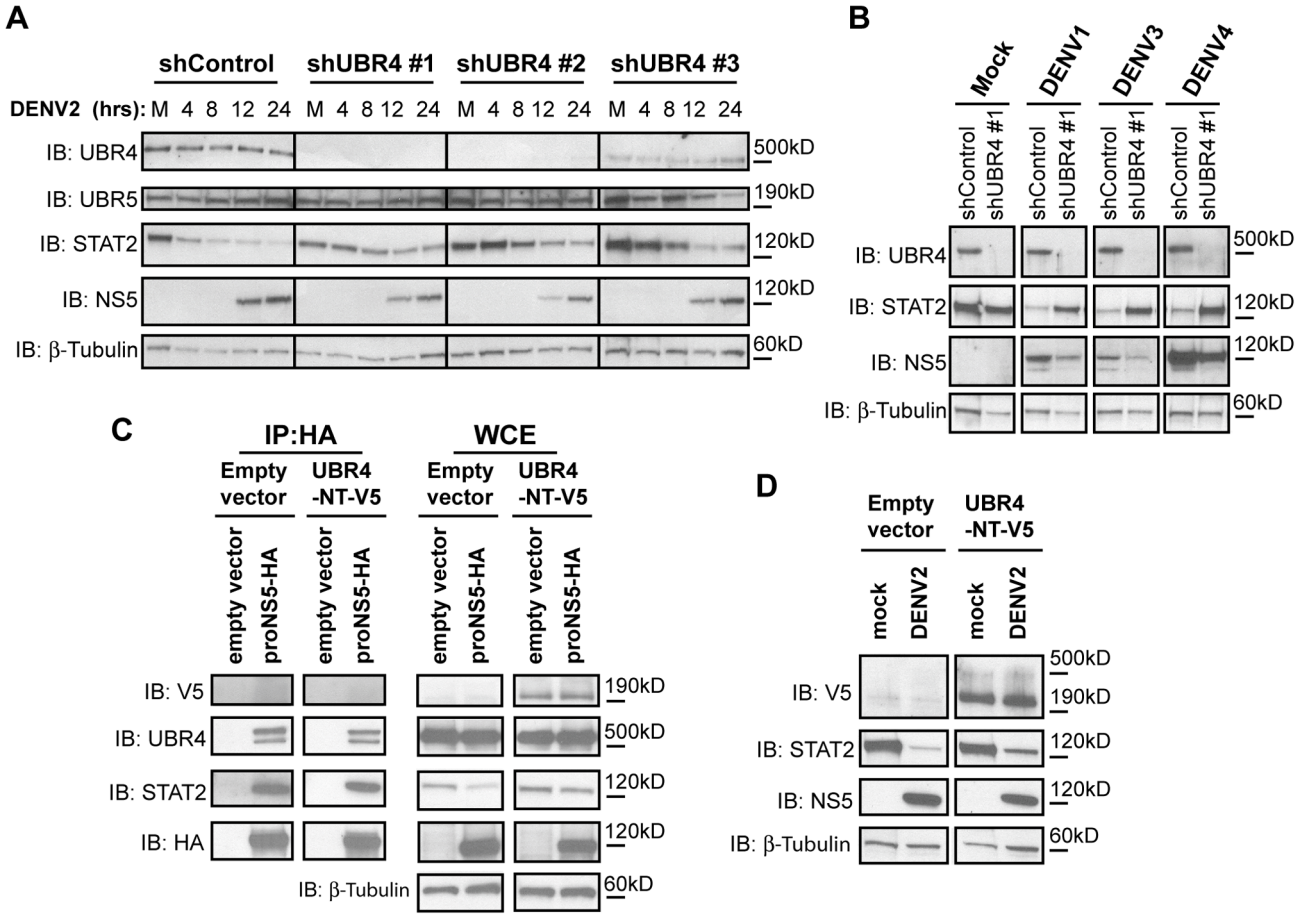
UBR4 promotes DENV-mediated STAT2 degradation. (A) 293T cells stably expressing non-targeting shRNA (shControl) or shRNA against UBR4 (shUBR4) were infected with DENV2 (Thailand/16681 strain) at an MOI of 10. Each shUBR4 clone expressed shRNA targeting different sequences within the UBR4 gene. Lysates from mock-infected cells (M) and cells that been infected with DENV2 for 4, 8, 12 or 24 hours were subjected to immunoblot (IB) analysis against UBR4, UBR5, STAT2, DENV NS5 and β-tubulin. (B) Lysates from shControl and shUBR4 293T cells that had been mock infected or infected with DENV1, DENV3 or DENV4 at an MOI of 1 for 24 hours were subjected to IB analysis. (C) A proteolytically-processed HA-tagged DENV NS5 construct or empty vector were transfected into 293T cells stably expressing a V5-tagged construct of the first 2233 amino acids of UBR4 (UBR4-NT). The lysates of these cells were subjected to anti-HA immunoprecipitation. Immunoblot (IB) analysis of the immunoprecipitate (IP) and whole cell extract (WCE) was performed against UBR4, human STAT2, V5, HA and β-tubulin. (D) 293T cells expressing UBR4-NT were mock infected or infected with DENV at an MOI of 1. Lysates were subjected to IB analysis against STAT2, V5, NS5 and β-tubulin at 24 hours post infection.

We next assessed the functional relevance of UBR4 in DENV-mediated STAT2 degradation. To test if UBR4 is required for DENV-mediated STAT2 degradation, UBR4 levels were stably reduced in 293T cells using small hairpin RNA (shRNA) directed against UBR4. Three stable UBR4-knockdown cell lines were generated using shRNA that targeted different sequences within UBR4. The cells were mock infected or infected with DENV2 at an MOI of 10, and lysed for western blot analysis at 4, 8, 12 and 24 hours post-infection. When cells expressing control non-targeting shRNA were infected with DENV2, STAT2 levels decreased by 4 hours post-infection. However, in the three independently-derived, UBR4-deficient 293T cell lines, STAT2 levels decreased at a slower rate (Figure 4A). Furthermore, NS5 levels were lower in the UBR4-knockdown cells than in the control cells suggesting that there was a DENV replication defect in UBR4-knockdown cells. The similar phenotype of the three UBR4-knockdown cell lines and their difference from the control cell line indicated that the effect of UBR4 knockdown on DENV-mediated STAT2 degradation was due to the decreased level of UBR4 and not to off-target effects of the shRNA. We next examined the functional relevance of UBR4 in mediating STAT2 degradation with the other three DENV serotypes (DENV1, 3 or 4). STAT2 levels were higher and NS5 levels were lower in shUBR4-expressing cells than in control cells (Figure 4B). Thus, UBR4 is required for efficient STAT2 degradation mediated by all four DENV serotypes.

The UBR4 gene is predicted to produce several splice variants encoding proteins of greater than 5000 amino acids. Since it is unclear which UBR4 isoform is required for DENV-mediated STAT2 degradation, we cloned a region of UBR4 (UBR4-NT) that is predicted to be present in all the large UBR4 isoforms and which also contains the UBR box, a 70-amino-acid zinc-finger-like domain required for recognition of N-end rule substrates [42]. The UBR box is located between amino acids 1662–1723 of the UBR4 reference sequence (NCBI Accession # Q5T4S7), and the UBR4-NT clone encodes amino acids 1–2233 of the reference sequence. Co-immunoprecipitation experiments revealed that proNS5-HA did not bind amino acids 1–2233 of UBR4 (Figure 4C) indicating that sequences in the C-terminal half of UBR4 are required to mediate its interaction with NS5. Also, expression of UBR4-NT had no effect on DENV-mediated STAT2 degradation (Figure 4D). The experiments in Figure 4 confirm that a functional UBR4-NS5-STAT2 complex is required for efficient STAT2 degradation and that multiple domains of UBR4 are required for this function.

### UBR4 enhances the replication of DENV in an IFN-I-dependent manner

**Figure 5:**
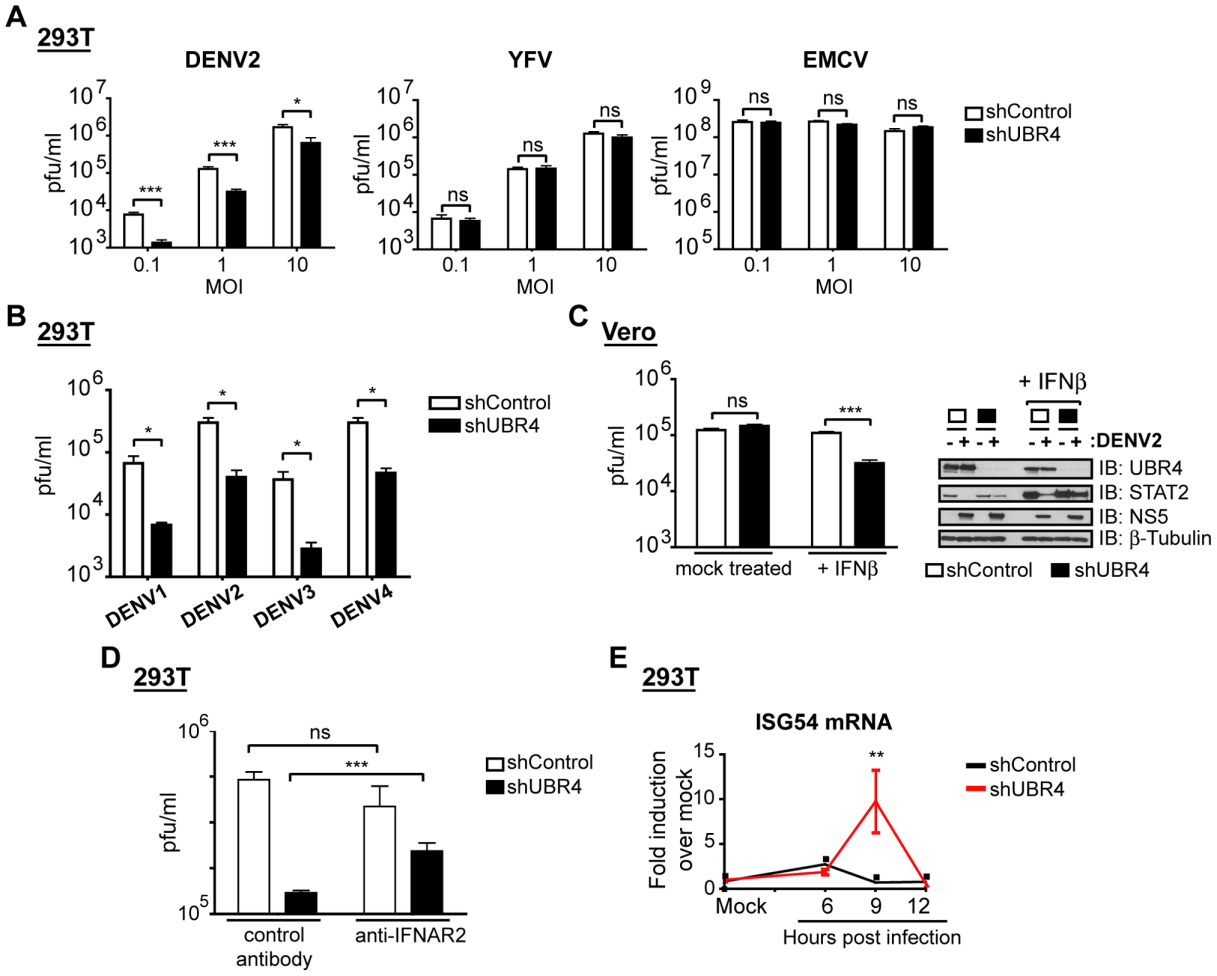
UBR4 enhances the replication of DENV in an IFN-I-dependent manner. (A) 293T cells stably expressing non-targeting shRNA (shControl) or shRNA against UBR4 (shUBR4 #2) were infected with DENV2 (Thailand/16681 strain), YFV (17D strain) or EMCV at the indicated multiplicities of infections (MOI). Cells and media were harvested at 24 hours post-infection and measured for virus by plaque assay on BHK-21 cells. (B) 293T cells were infected with DENV1, 2, 3 or 4 at an MOI of 1 and the virus levels were measured by plaque assay on BHK-21 cells 24 hours later. (C) Vero cells stably expressing non-targeting shRNA (shControl) or shRNA against UBR4 (shUBR4 #1) were infected with DENV2 (Thailand/16681 strain) at an MOI of 0.1. Cells were mock-treated or treated with IFN-I (1000units/ml IFNβ) at 6 hours post infection. Cells and media were harvested at 24 hours post-infection for plaque assay or immunoblotting (IB) with antibodies against UBR4, STAT2, NS5 and β-tubulin. (C) 293T cells were infected with DENV2 at an MOI of 1 and incubated with media containing 0.1 µg/ml control IgG antibody or 0.1 µg/ml IFNAR antibody. Cells and media were harvested at 24 hours post-infection and measured for virus by plaque assay on BHK-21 cells. (D) 293T clones were infected with DENV2 at an MOI = 1 and their RNA harvested at the indicated time post infection. The levels of ISG54 mRNA were measured by qPCR, normalized to 18s (a housekeeping gene) and represented as fold induction over mock samples.). Each graph represents the mean +/− the standard deviation of 6 experiments (A), 3 experiments (B, C and D), or 2 experiments (E). Statistical analyses were conducted using the unpaired t test function of Prism 4 for Macintosh (GraphPad Software, USA). *p<0.05; **p<0.01; *** p<0.001; ns = not statistically significant, where p>0.05.

The ability of DENV to degrade STAT2 determines how well it replicates in an IFN-I-competent cell [44]. Thus, a protein that is required for DENV-mediated STAT2 degradation should also enhance DENV replication in IFN-I-competent cells. To test if UBR4 is required for DENV replication, UBR4-knockdown 293T cells were infected with DENV2 at multiplicities of infection (MOI) of 0.1, 1 and 10, and measured for virus at 24 hours post-infection. DENV replicated to lower levels in UBR4-knockdown cells than in control cells (Figure 5A). The replication defect was most striking at a lower MOI and an approximately 10-fold decrease in virus levels was observed in shUBR4 cells with an MOI of 0.1 of DENV. In contrast, UBR4 depletion had no effect on the replication of YFV or encephalomyocarditis virus (EMCV), a positive-strand RNA virus belonging to the *Picornaviridae* family, indicating a specific requirement of UBR4 in DENV replication (Figure 5A). DENV1, 3 and 4 also replicated to lower levels in UBR4-knockdown cells than in control cells indicating that UBR4 is required for the efficient replication of all four DENV serotypes (Figure 5B).

Since UBR4 was required for DENV-mediated STAT2 degradation, we hypothesized that the DENV replication defect in UBR4-deficient cells was due to an inability of DENV to antagonize IFN-I signaling by degrading STAT2. If this is the case, lack of IFN-I should compensate for the requirement of UBR4 in DENV replication. To test this, we infected control and UBR4-knockdown Vero cells with DENV. Vero cells lack IFN-I genes and therefore cannot make IFN-I in response to viral infection [45]. DENV replicated to similar levels in UBR4-knockdown and control Vero cells (Figure 5C). Yet when Vero cells were infected with DENV and then exogenously treated with IFN-I 6 hours later, a DENV replication defect was observed in the UBR4-deficient Vero cells (Figure 5C). Protein levels of NS5, UBR4, and STAT2 in UBR4-knockdown Vero cells showed that UBR4 levels were indeed lower and that DENV-mediated STAT2 degradation was defective in UBR4-knockdown cells (Figure 5B).

Treating DENV-infected UBR4-knockdown 293T cells with a neutralizing anti-IFNAR antibody corroborated the effect of IFN-I on DENV replication in UBR4-deficient Vero cells (Figure 5D). We observed a significant increase in DENV replication in UBR4-knockdown 293T cells treated with the neutralizing anti-IFNAR antibody compared to UBR4-knockdown 293T cells treated with IgG control antibodies. This contrasts with what was observed in control 293T cells where DENV replication was unaffected by treatment with anti-IFNAR antibodies (Figure 5D).

IFN exerts its biological effect by upregulating interferon-stimulated genes (ISGs), which encode products that restrict viral replication. To examine the biological relevance of UBR4 in preventing the antiviral action of IFN-I during DENV infection, we examined the induction of ISG54 mRNA in UBR4-knockdown 293T cells. There was a significant induction of ISG54 mRNA in UBR4-knockdown cells during DENV infection compared to control cells (Figure 5E). These results indicate that UBR4 is required for preventing the antiviral action of IFN-I during DENV infection.

**Figure 6:**
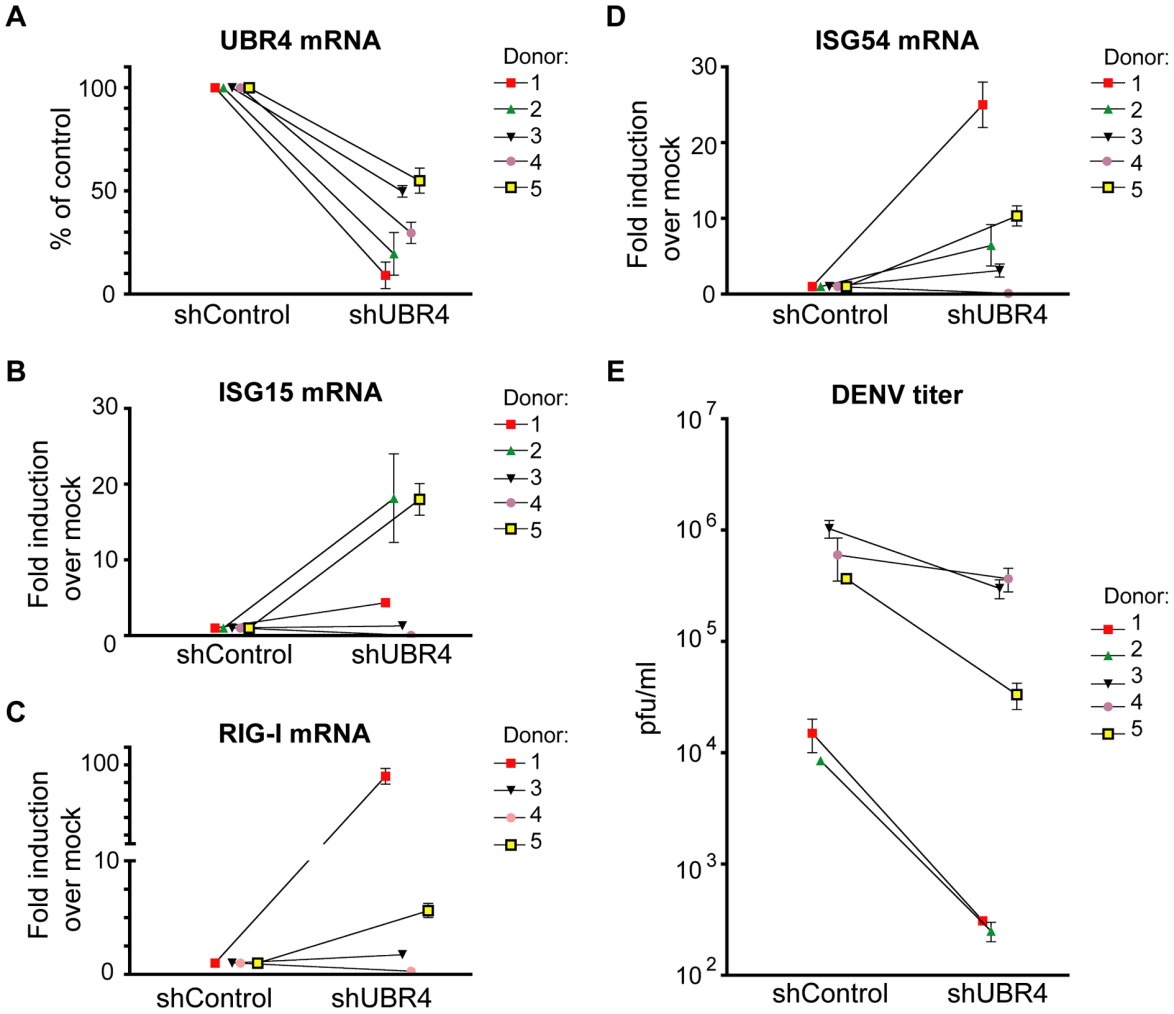
UBR4 enhances the replication of DENV in monocyte-derived dendritic cells. Monocytes from 5 donors were transduced with a non-targeting shRNA lentivirus (shControl) or an shRNA lentivirus against UBR4 (shUBR4 #2). Monocytes were differentiated for 5 days to monocyte-derived dendritic cells (MDDCs) before infecting with DENV2 (Thailand/16681 strain) at an MOI of 3. Cells were harvested at 12 hours post infection for measurement of (A) UBR4, (B) ISG15, (C) RIG-I, and (D) ISG54 mRNA levels, or (E) supernatants were harvested at 48 hours post infection for measurement of virus levels via plaque assay. RIG-I mRNA was undetectable in 1 donor. Statistical analyses were conducted using the two-way ANOVA function of Prism 4 for Macintosh (GraphPad Software, USA). p<0.001 for UBR4, ISG15, RIG-I and ISG54 levels and p<0.01 for DENV titers in shControl versus shUBR4 cells.

Dendritic cells are thought to be an important cell type in which DENV replicates *in vivo* [46], [47]. We reduced UBR4 levels in primary MDDCs from five donors using shRNA lentiviral constructs, and tested the effect of this decrease on DENV replication. When MDDCs from each donor were infected at an MOI of 3, approximately 35% of transfected cells were highly infected and showed viral glycoprotein (E) expression by FACS. At 12 hours post infection, as expected, the levels of UBR4 were decreased in the UBR4-knockdown cells compared to control cells (Figure 6A). When the levels of ISG15, RIG-I and ISG54 mRNA were analyzed in these cells, more ISGs were induced in four of the five donors (Figure 6B, 6C and 6D respectively). In addition, more DENV was present in control cells than in UBR4-knockdown cells at 48 hours post infection (Figure 6E). Thus, UBR4 is required for inhibiting ISG induction and increasing DENV replication in a primary cell type that is of importance in DENV infections.

## Discussion

The IFN-I response is one of the first lines of protection against DENV infection, and serves to curb viral replication and dissemination by generating an antiviral intracellular environment [48]. The potency of the type I IFN pathway is exemplified by the fact that DENV antagonizes both IFN synthesis and IFN signaling in order to ensure its replication and survival [13], [14], [33], [34], [35], [36], [37], [38]. DENV NS5 inhibits IFN-I signaling by mediating proteasome-dependent STAT2 degradation, and STAT2 degradation promotes DENV replication [13], [14], [44]. With this study, we report the discovery of a host factor, UBR4, that is essential for DENV-dependent STAT2 degradation. We describe the interaction of UBR4 with NS5 and show that this interaction is crucial for inhibiting type-I IFN signaling and promoting efficient DENV replication.

UBR4 associates with DENV NS5 but not with the closely related YFV NS5 or WNV NS5. UBR4 also binds preferentially to proteolytically-processed DENV NS5, which is the form of NS5 that efficiently mediates STAT2 degradation. Binding of UBR4 to DENV NS5 requires amino acids T2 and G3 of NS5, which are also critical for STAT2 degradation. These amino acids are conserved amongst the four DENV serotypes but are absent in other flaviviruses (Figure 2F). Though NS5 is the most highly conserved flavivirus protein, the high degree of specificity exhibited by UBR4 for DENV NS5 underscores the differences between the various flaviviral NS5 proteins.

In 293T cells and primary human dendritic cells, DENV replicates best when UBR4 levels are normal, but when UBR4 levels are reduced, DENV-mediated STAT2 degradation is reduced and DENV replication decreases as a consequence (Figures 5 and 6). In Vero cells, which do not produce IFN-I [45], UBR4 depletion does not affect DENV replication unless these cells are treated with exogenous IFN-I (Figure 5C). Furthermore, the DENV replication defect caused by UBR4 knockdown in 293T cells can be decreased by treating the cells with antibodies that block the IFN-I receptor and decrease IFN-I signaling (Figure 5D). The DENV replication defect seen in UBR4-knockdown 293Ts and MDDCs can be explained by an increase in ISG levels in DENV-infected UBR4-knockdown cells versus DENV-infected control cells (Figure 5E and Figure 6). Thus, in the absence of IFN-I, there is no need for DENV to antagonize IFN-I signaling and cellular levels of UBR4 are irrelevant for DENV replication. However, upon activation of the IFN-I signaling pathway, UBR4 becomes necessary for DENV replication. Reducing STAT2 levels is essential for DENV to preempt the establishment of a cellular antiviral state, thus ensuring its efficient replication.

Antagonism of IFN signaling is one of the factors responsible for the limited host tropism of DENV to human and nonhuman primates. DENV does not replicate to high levels or induce disease in IFN-competent mice [44], [49]. Our previous results indicated that the cellular machinery needed for DENV replication in murine cells is in place but is limited by the inability of NS5 to associate with murine STAT2 and inhibit murine IFN-I signaling [44]. Other blocks such as the type II IFN pathway also diminish DENV replication in mice, but the IFN-I signaling pathway restricts early replication [44], [50]. Here we show that DENV NS5 associates with murine UBR4 in murine cells. This is in keeping with our previous results [44], and suggests that the development of a genetically-modified mouse that expresses a functional human STAT2 in place of its murine counterpart should allow increased DENV replication. We predict, therefore, that DENV NS5 will mediate human STAT2 degradation in these mice by co-opting mouse UBR4. Such a mouse might provide the basis for the development of an immune-competent mouse model of DENV infections.

The 600 kDa large UBR4 is highly conserved and found in organisms as diverse as mammals, insects, plants and worms. It belongs to the N-recognin family, which contains proven and predicted E3 ligases that recognize and degrade proteins containing destabilizing N termini. The seven members of the UBR family, UBR1 to UBR7, encode a 70-amino-acid zinc finger motif known as the UBR box, which is necessary for substrate recognition [42]. The better-characterized members of the UBR family are UBR1, UBR2 and UBR5. UBR1 and UBR2 are RING domain-containing N-recognins, which recognize N-end rule substrates and target them for degradation [42]. UBR1 and UBR2 are also involved in N-end-rule-independent quality control protein degradation [51]. UBR5 is a HECT-domain containing E3 ligase that binds N-end rule substrates [42], but can also target non-N-end rule substrates like E6AP for degradation [52]. UBR4 contains neither a HECT nor a RING domain.

A dearth of UBR4 literature exists because of the difficulty that manipulating the UBR4 gene presents. The UBR4 gene contains 106 exons, and produces multiple splice variants that conceivably have different functions. UBR4 forms a chromatin scaffold when bound to retinoblastoma protein (Rb) in the nucleus, and it also influences cytoskeleton organization by binding clathrin in the cytoplasm [53]. Both of these are structural roles for which no N-end rule or other E3 ligase activities have been detected. A second virus, human papilloma virus, is known to exploit UBR4's role in cellular morphology to initiate anchorage-independent growth and cellular transformation [54], [55]. Although UBR4 is part of a family of UBR E3 ligases involved in the N-end rule pathway, the involvement of the N-end rule in the NS5-dependent degradation of STAT2 seems unlikely. Our group has previously demonstrated that the identity of NS5's first residue is not relevant for STAT2 degradation as long as the precursor is correctly processed [13]. In addition, we show that residues T2 and G3 of NS5 are critical for binding to UBR4 and for mediating STAT2 degradation, but they are considered to be stabilizing residues within the N-end rule. This does not exclude UBR4 from having E3 ligase activity that is independent of the N-end rule. Though it lacks an obvious catalytic domain such as the HECT or RING domains, UBR4 contains a cysteine-rich domain (CRD) that is unique to the UBR4 group. It is currently unknown if CRD functions as a ligase domain. Our experiments with the N-terminal region of UBR4 suggest that domains from the C terminus, which contain the CRD, are necessary for its function in DENV-mediated STAT2 degradation.

**Figure 7:**
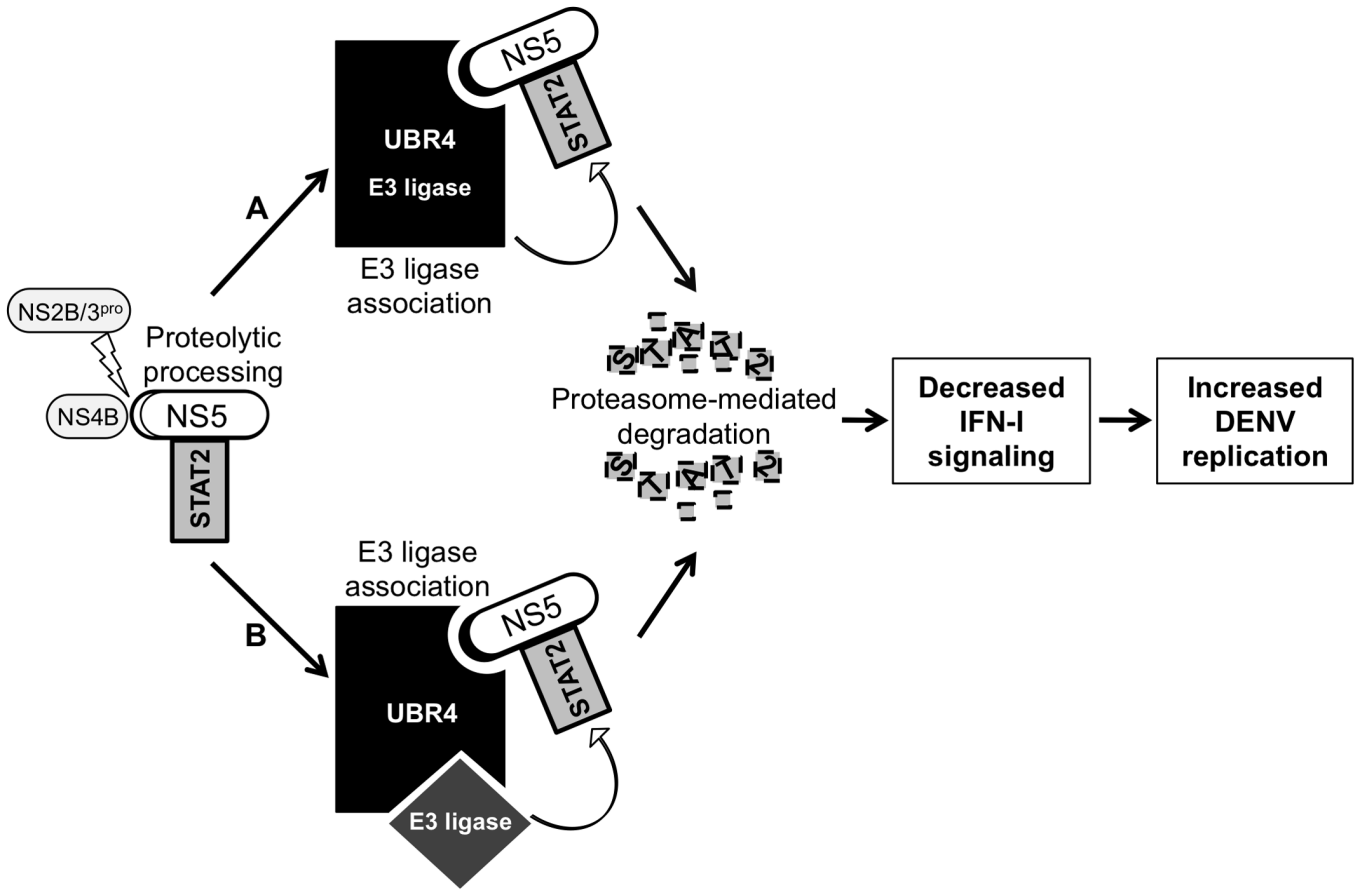
Model of DENV-mediated STAT2 degradation. NS2B/3 protease cleaves NS4B away from NS5 to yield proteolytically-processed NS5. Proteolytically-processed NS5 brings STAT2 into contact with the degradation machinery of the cell by binding UBR4. UBR4 may function as an E3 ligase (A), but as it lacks a known E3 ligase catalytic domain such as the HECT or RING, it may instead act as the recognition component or scaffold of an E3 ligase complex (B). STAT2 degradation prevents the establishment of an antiviral state thereby allowing DENV to replicate optimally.

Finally, we propose two working models: one based on the hypothesized UBR4 E3 ligase catalytic activity, and another which postulates a scaffolding role for UBR4 based on its described interactions with clathrin and retinoblastoma protein (Figure 7). Efforts are currently being made to clone and express the predicted UBR4 isoforms so as to further evaluate the function of UBR4 in DENV-mediated STAT2 degradation, and to explore its potential as a target for rationally-designed DENV therapeutics.

## Materials and Methods

### Cell lines and viruses

293T, Hepa1.6, U6A, BHK and Vero cells were maintained in DMEM (Life Technologies) supplemented with 10% fetal bovine serum (Life Technologies) and 1% penicillin/streptomycin mix (Life Technologies). C6/36 cells were maintained in RPMI 1640 medium supplemented with 10% fetal bovine serum (Life Technologies). Hepa1.6 cells were kindly provided by Matthew Evans (Mount Sinai School of Medicine, New York, NY). U6A cells were a kind gift of George Stark (Lerner Research Institute, Cleveland, OH) and were previously described [43].

High-titer stocks of DENV1, DENV2, DENV3, DENV4, yellow fever virus (YFV-17D) and encephalomyocarditis virus (EMCV) were obtained by passage in C6/36 cells, BHK cells, and Vero cells, respectively.

### Plasmids and transfections

pCAGGS-CTAP was a kind gift from Luis Martinez-Sobrido (University of Rochester). A gene cloned into pCAGGS-CTAP produces a fusion protein that is C-terminally tagged with a TAP tag: calmodulin binding protein followed by two tobacco etch virus (TEV) cleavage sites followed by a protein A tag. The sequences of the primers used for the construction of the RFP-ubiquitin-NS5 fragment that was cloned into pCAGGS-CTAP are available upon request. The primers sequences used for cloning UBR4-NT (1–2233 of UBR4, NCBI Accession Q5T4S7) into pCDNA6 are also available upon request. All other viral gene expression constructs were cloned into pCAGGS-HA and were described previously [13], [15]. The Flag-tagged STAT1, STAT2 and chimeric STAT constructs were previously described [44].

All cells were transfected using Lipofectamine 2000 (Invitrogen) according to the manufacturer's protocol. 293T cells were transfected at a ratio 1∶2 (µg plasmid DNA: µL Lipofectamine 2000) while Vero, Hepa1.6 and U6A cells were transfected at a ratio 1∶3.

### Tandem affinity purification and immunoprecipitation

Cells were lysed for tandem affinity purification (TAP) or immunoprecipitation two days post transfection. For tandem affinity purification, cells were lysed in TAP buffer (25% glycerol, 50 mM Tris HCL pH 8, 0.5% NP40, 200 mM NaCl, 1 mM β-mercaptoethanol, protease inhibitor cocktail (Roche). Lysates were spun at 15,000 g for 10 minutes and the supernatant was incubated with IgG beads (Roche) for 4 hours then washed with TAP buffer. The beads were then incubated with TEV buffer (TAP buffer containing 0.5 mM EDTA, 1 mM DTT units) and 50 units AcTEV enzyme (Invitrogen) overnight. The beads were spun at 15,000 g for 10 minutes then the supernatant was applied to calmodulin beads (Roche) in a calmodulin bead (CB) buffer (TAP buffer containing 4 mM CaCl_2_ and 2 mM imidazole) for 8 hours, then washed in CB buffer. The protein was eluted from the calmodulin beads by boiling for five minutes in Laemmli sample buffer (BioRad).

For immunoprecipitation, cells were lysed in TAP buffer then incubated for two hours with anti-FLAG or anti-HA beads (#F2426 and #E6779 respectively, Sigma-Aldrich) or for 4 hours with rabbit anti-STAT2 antibody (Santa Cruz) or mouse anti-GFP antibody (Abcam) followed by 2 hours of protein A-agarose (Roche) or protein G-agarose (Roche), respectively. The beads were washed with TAP buffer then the protein was eluted from the beads by boiling for five minutes in Laemmli sample buffer (BioRad).

### Western blot analysis of transfected and infected cells

Proteins lysates were boiled with Laemmli sample buffer and resolved on 4–15% or 7.5% gels (BioRad) and then transferred to PVDF membrane (Millipore) by standard methods. Membranes were blocked with 3% BSA in TBS-Tween (20 mM Tris-HCl, pH 7.4; 150 mM NaCl; 1% Tween) and then incubated with antibodies and subjected to western blot. Benchmark Protein Ladder (Invitrogen) was used to depict the size of protein bands. The primary antibodies used in this study were: rabbit anti-human STAT2 (sc-476, Santa Cruz), rabbit anti-mouse STAT2 (4597, Cell Signaling), rabbit anti-STAT1 (610120, BD Biosciences), mouse anti-β-tubulin (T0198, Sigma-Aldrich), mouse anti-HA (H9658, Sigma-Aldrich), mouse anti-Flag (F3165, Sigma-Aldrich), mouse anti-V5 (R960-25, Invitrogen), rabbit anti-UBR4 (ab86738, Abcam), HRP-linked anti-GAPDH (ab9385, Abcam), rabbit anti-UBR5 (ab70311, Abcam), and rabbit anti-NS5 [13]. The secondary antibodies used in this study were HRP-linked anti-mouse IgG (#NA931V, GE Healthcare) and HRP-linked anti-rabbit IgG (#NA934V, GE Healthcare). Where indicated, quantification of western blots was done by using Image J to compare the ratio of UBR4 (seen as two bands or one band based on the resolution of the tris-glycine gel used) to NS5.

### Immunofluorescence

To analyze the intracellular localization of endogenous UBR4 and DENV NS5, Vero cells that had been grown on glass cover slips were transfected with 1 µg of the indicated plasmids. After 24 hours post infection, cells were fixed and permeabilized for 30 minutes with ice cold methanol acetone (1∶1, v/v) and 0.5% NP-40, then washed with PBS. Following PBS washes, cells were blocked in blocking buffer (0.2% cold waterfish gelatin (Sigma-Aldrich, USA) and 0.5% BSA in PBS) for 1 hour at room temperature (RT), and stained with primary antibodies (anti-UBR4 at a 1∶100 dilution, and anti-HA at a 1∶1000 dilution) overnight at 4°C. The cells were washed in PBS and incubated with secondary antibodies to Alexa Fluor 488 and Alexa Fluor 555 (Invitrogen, USA) at 1∶500 dilution in blocking buffer for 1 hour at RT. Nuclear chromatin staining was performed by incubation in blocking solution containing 0.5 mg/ml 4′,6-diamidino-2-phenylindole, DAPI (Sigma-Aldrich). Cells were washed and coverslips mounted using Prolong antifade reagent (Invitrogen). Images were captured using a Leica SP5-DM confocal microscope at the Microscopy Shared Research Facility at Mount Sinai School of Medicine.

### Establishment of knockdown cell lines with lentiviruses expressing shRNA

The 293T and Vero cell lines stably expressing non-silencing shRNA or shRNA against UBR4 were made by infecting cells with shRNA-encoding lentiviruses (according to the manufacturer's protocol) and selecting cells with puromycin (1 µg/ml for 293T cells and 5 µg/ml for Vero cells) for two weeks before DENV, YFV or EMCV infection.

The lentiviruses used to make 293T shUBR4 clones were purchased from Open Biosystems. Lentivirus 1 (Clone ID: V3LHS_318553; target sequence: CGCTTCGACTTCATGCTCT) targets nucleotides 11132–11150 of UBR4. Lentivirus 2 (Catalog #: V3LHS_318554; target sequence: CGGATCAGCTCCTATGTCA) targets nucleotides 3140–3158 of UBR4. Lentivirus 3 (Catalog #: V3LHS_318555; target sequence: AGGTTTTTGTCTACAATGA) targets nucleotides 2357–2375 of UBR4. The non-silencing control lentivirus was catalog number RHS4348. The Vero shUBR4 clone was made using lentivirus 1, while the Vero shControl was made using non-silencing control lentivirus.

### Infection of 293T and Vero cells

Cells were infected at the indicated multiplicity of infection (MOI) and maintained in DMEM with 10% FBS. For exogenous IFN-I treatment of Vero cells, 1000 units/ml IFNβ (PBL Interferon Source) were added at 6 hours post infection. For western blotting, cells were lysed with TAP buffer at each time point and the lysates were clarified by centrifugation then boiled in Laemmli sample buffer. For virus titration, cells and media were frozen at each time point and clarified by centrifugation. DENV and YFV titers were measured by plaque assay on BHK-21 cells, and EMCV titers were measured by plaque assay on Vero cells.

### Isolation, transduction and stimulation of MDDCs

Peripheral blood mononuclear cells were isolated from buffy coats of healthy human donors by Ficoll density gradient centrifugation (Histopaque, Sigma Aldrich) as previously described [33]. Buffy coats were obtained from the Mount Sinai Blood Donor Center and New York Blood Center. Briefly, CD14+ cells were purified using anti-human CD14 antibody-labeled magnetic beads and iron-based MiniMACS LS columns (Miltenyi Biotech). After elution from the columns, 2×10^5^ cells were plated in 96-well plates and transduced with VSV-G pseudo-typed SIV VLPs (pSIV3+, an SIV gag-pol expression plasmid containing Vpx) and lentiviral control or UBR4-specific shRNA vectors for 3 hours by spinoculation in the presence of 2 µg/mL polybrene (Sigma) with sufficient viruses to transduce >95% of the cells. Subsequently, cells were washed, resuspended in DC medium (RPMI medium [Invitrogen], 10% fetal calf serum [HyClone], 100 U/ml penicillin, and 100 µg/ml streptomycin [Invitrogen]) supplemented with 500 U/ml human granulocyte-macrophage colony-stimulating (Peprotech), and 1,000 U/ml human interleukin-4 (hIL-4; Peprotech), and incubated for 5 days at 37°C. At 5 days post transduction, MDDCs were either mock infected or infected with DENV2 at an MOI of 3. At 12 hours post infection (hpi) cells were harvested for qPCR analysis, and at 48 hpi supernatants were collected for titration of virus levels by plaque assay on BHK-21 cells. Cells were also harvested for cytometry analysis.

### Quantitative RT-PCR (qPCR)

Total RNA was isolated from samples using the RNeasy kit (Qiagen) and subjected to DNase digestion with Turbo DNase (Ambion). Reverse transcription was performed using the high capacity cDNA reverse transcription kit (Applied Biosystems). qPCR was performed in 384-well plates in triplicates using SYBR green I master mix (Roche) in a Roche LightCycler 480. Relative mRNA values were calculated using the ΔΔCt method using 18S rRNA as internal control and plotted as fold change by normalizing to mock-control samples.

UBR4 qPCR primers: Forward = GGTGTTCCAGAGGCTAGTGATC; Reverse = CCAACTGCTTCTGCGGTTCCTT

ISG15 qPCR primers: Forward = TCCTGGTGAGGAATAACAAGGG; Reverse = GTCAGCCAGAACAGGTCGTC

RIG-I qPCR primers: Forward = GGCATGTTACACAGCTGACG; Reverse = TGCAATATCCTCCACCACAA

ISG54 qPCR primers: Forward = ATGTGCAACCTACTGGCCTAT; Reverse = TGAGAGTCGGCCCATGTGATA

18S RNA qPCR primers: Forward = GTAACCCGTTGAACCCCATT; Reverse = CCATCCAATCGGTAGTAGCG

### Flow cytometry

DENV-infected DCs were fixed and permeabilized with Cytofix and Cytoperm reagent (BD Pharmingen) according to the manufacturer's recommendations. Then, cells were stained with 4G2 (ATCC), a mouse monoclonal antibody specific for the E protein, as a primary antibody and a FITC-labeled anti-mouse antibody as a secondary antibody. Flow cytometry was performed using a FACScan flow cytometer (Becton Dickinson) and analyzed with FlowJo software.
